# Perceived Opportunities for Physical Activity and Willingness to Be More Active in Older Adults with Different Physical Activity Levels

**DOI:** 10.3390/ijerph18116146

**Published:** 2021-06-07

**Authors:** Eeva Aartolahti, Johanna Eronen, Timo Törmäkangas, Taina Rantanen, Mirja Hirvensalo, Lotta Palmberg, Heidi Skantz, Anne Viljanen, Erja Portegijs, Susanne Iwarsson, Merja Rantakokko

**Affiliations:** 1 Faculty of Sport and Health Sciences, University of Jyväskylä, FI-40014 Jyväskylä, Finland; johanna.eronen@jyu.fi (J.E.); timo.tormakangas@jyu.fi (T.T.); taina.rantanen@jyu.fi (T.R.); mirja.hirvensalo@jyu.fi (M.H.); lotta.m.palmberg@jyu.fi (L.P.); heidi.e.leppa@jyu.fi (H.S.); anne.viljanen@jyu.fi (A.V.); erja.portegijs@jyu.fi (E.P.); 2 Institute of Rehabilitation, JAMK University of Applied Sciences, FI-40100 Jyväskylä, Finland; merja.rantakokko@jamk.fi; 3 Gerontology Research Center, Faculty of Sport and Health Sciences, University of Jyväskylä, FI-40014 Jyväskylä, Finland; 4 Department of Health Sciences & Centre for Ageing and Supportive Environments (CASE), Lund University, SE-22100 Lund, Sweden; susanne.iwarsson@med.lu.se

**Keywords:** equity, unmet need, physical function, aging, exercise

## Abstract

This study examined equity in physical activity (PA) by investigating whether perceived opportunity for PA was associated with willingness to be more active. Among community residents (75, 80, or 85 years old, *n* = 962) perceived opportunity for PA (poor and good), willingness to be more active (not at all, a bit, and a lot), and level of PA (low, moderate, and high) were assessed via questionnaires. Multinomial logistic regression showed that physical activity moderated the association between poor opportunity and willingness to increase PA. Among those with moderate PA, poor opportunity for PA increased the odds of willingness to be a lot more active (multinomial odds ratio, mOR 3.90, 95% confidence interval 2.21–6.87) than not wanting to be more active compared to those perceiving good opportunities. Associations were similar at high PA levels (*p* < 0.001), but were not found at low PA levels. Those with moderate or high PA wish to increase their activity particularly when the perceived opportunities for activity are not optimal. Among those with low PA, perceived opportunities are not associated with a perceived need to increase physical activity. Increasing equity in physical activity in old age requires provision of support and opportunities at every level of physical activity.

## 1. Introduction

Increasing equity in opportunities for physical activity in old age is an important target of sport and health policy, and improving opportunities to engage in physical activity at different stages of functional limitations is also in line with physical activity recommendations [1]. If we can better understand the interrelationships between willingness for physical activity and perceived opportunities for it among older persons with different levels of physical activity, we will have better prerequisites to find more optimal solutions for physical activity that meets the different needs of the aged population.

As people age, their level of physical activity often declines and many engage in lighter physical activities [2]. For older persons, physical activity often accumulates while running daily errands such as going shopping and doing domestic chores [3]. Physical activity level may be dependent on urban design and infrastructure of neighborhoods as it has been shown that older adults are more active when living in neighborhoods with high walkability [4], residential density [5,6], and access to public transportation [7]. Maintaining physical activity in the older age groups is important for sustaining functional ability, independent living, and community participation [2,8,9].

Not only because health benefits of physical activity, but also from a health equity perspective, all those who are willing to be physically active should have the opportunity for it [10], regardless of age and functional ability [11]. Some policy actions, such as the Active Ageing Policy by the World Health Organization (WHO), have targeted this issue [12] and continuing participation in activities with a wide range of tasks is also a goal in the Global Strategy and Action Plan on Ageing and Health [13]. WHO’s Active Ageing Policy emphasizes optimizing opportunities for health and participation to enhance quality of life as people age. However, despite policy actions and recognizing the importance of physical activity participation among older people, not all older people are able to engage in physical activities at the desired form, amount, or intensity [14].

In the physical activity context, the ability to engage in physical activities in the way that one wants, constitutes autonomy [15]. Perceived autonomy entails the personal choice of whether an individual is willing to be physically active or not [15]. Autonomy is one of the basic psychological needs, along with competence and relatedness. Satisfaction of the basic psychological need leads to positive outcomes, such as higher quality of life and improved physical health [10].

While physical activity is recommended to everyone, it is important to remember that even when an individual is willing to be physically more active, not all have the opportunity for it. Satisfying the need for physical activity, thus, comes from the willingness for physical activity, but also from perceiving opportunity for it. Unmet physical activity need describes the difference between the desired and true state of physical activity. It is a situation in which a person would like to be more physically active but perceives no opportunity for it. It is a felt need, meaning that it describes what a person wants but feels left out from [16]. Unmet physical activity need, when present, describes the individual’s perception of his or her current status. In one study, 14% of older Finnish participants reported experiencing unmet physical activity need [17]. In cross-sectional observations, unmet physical activity need has been identified among older people with mobility limitations, depressive symptoms, musculoskeletal diseases [17], lower socioeconomic status [18], and poorer quality of life [19]. Among older persons who did not report unmet physical activity need at baseline, lower physical activity levels and lower step counts preceded its development over a two-year follow-up [20].

Increasing knowledge about equity in physical activity warrants a more detailed investigation on perceived opportunity relative to willingness for physical activity. Unmet physical activity need was previously assessed with two dichotomous questions measuring the willingness and perceived opportunities to increase physical activity [17,21]. However, this measurement method only assessed whether a person experiences unmet physical activity need or not, and failed to provide more detailed information about those not reporting unmet physical activity need. For example, we do not know, if a person would be satisfied with only a small increase in daily activity, and whether they perceive minor or major limitations in their opportunities. Moreover, it would be important to know if the perceived need manifests similarly in inactive persons who rarely move outside the home and those who lead an active lifestyle in wider areas.

In order to address equity in physical activity, it is necessary to not only recognize those older adults who experience unmet physical activity need, but also understand different situations when physical activity is perceived as being inadequate. We need to know whether older persons wishing to increase their level of physical activity perceive that they have the opportunity to be more active, and also identify those persons who, despite perceiving good opportunities for physical activity, report low physical activity. More importantly, we need to identify those older individuals who have become marginalized from physical activity against their wishes.

The aim of this study was to analyze whether perceived opportunity for physical activity is associated with willingness to increase physical activity and current physical activity levels and whether the association between willingness and opportunity is similar at different levels of physical activity. 

## 2. Materials and Methods

### 2.1. Study Sample and Data Collection

For this cross-sectional observational study, we used data from the Active aging—resilience, and external support as modifiers of the disablement outcome (AGNES) research project. The inclusion criteria were age of 75, 80, or 85 years and living independently in the study of Jyväskylä in central Finland [22]. City of Jyväskylä has urban and walkable environment and all participants lived within a 10-km radius of the city center or within reach of local public transportation. Exclusion criteria were unwillingness to participate and inability to communicate. The recruitment interval was September 2017 to December 2018.

Detailed information on the AGNES protocol and study flow was described in previous papers [22,23]. An initial information letter inviting participants was sent to 2348 persons, of whom 1018 took part in face-to-face home interviews, 1004 responded to the postal questionnaire, and 910 participated in the laboratory assessments at the research center. The final sample for the present study included 962 participants (96%) who responded to the questions regarding physical activity and the willingness to increase opportunities for physical activity in the postal questionnaire. In addition to these questions, the postal questionnaire included questions about health, medications, falls, and social contacts. All participants were Caucasian and Finnish speaking.

### 2.2. Measurements

Development of a questionnaire to measure the level, opportunities, and willingness of physical activity as well as test–retest results are described in detail in Appendix A. The questions are briefly described here.

Level of physical activity was assessed with the question [24] “Which of the following options describes your current physical activity best? Include exercise and habitual physical activity, such as walking to a store and cycling. Choose one option.”

1. I do not do more activities than necessary to perform daily chores.
2. I do light activities 1–2 times per week (for example, home gymnastics, slow walking, or outdoor activities).
3. I do light activities several times per week (for example, home gymnastics, slow walking, or outdoor activities).
4. I do activities 1–2 times per week to the point of some increase in breathing and perspiring (for example, brisk walking, indoor activities, or swimming).
5. I do activities several times per week to the point of some increase in breathing and perspiring (for example, brisk walking, indoor activities, or swimming).
6. I do activities that lead to quite a large increase in breathing and perspiring or participate in competitive sports.

In the analyses, alternatives 1 and 2 (low level), 3 and 4 (moderate level), and 5 and 6 (high level) were combined due to low response frequencies in the most extreme categories.

Willingness to increase physical activity was assessed with the question “Would you like to increase your level of physical activity?” with the following response options:

1. Yes, I would like to be very much more active than I currently am.
2. Yes, I would like to be much more active than I currently am.
3. Yes, I would like to be a bit more active than I currently am.
4. No, I would not like to be more active than I currently am.

In the analyses, alternatives 1 and 2 (a lot more active) were combined due to low response frequencies in the most extreme category. Alternative 3 (a bit more active) and 4 (not more active) were used as independent categories.

Perceived opportunity to physical activity was asked with the question “How do you perceive your opportunities for physical activity? Include exercise and habitual physical activity, such as walking to a store or cycling. Response options:

1. Very good
2. Good
3. Moderate
4. Poor
5. Very poor/not possible

In the analyses, alternatives 1 and 2 (good) as well as 3, 4, and 5 (poor) were combined due to low response frequencies.

### 2.3. Descriptive Variables

The Mini Mental State Examination (MMSE) [25] was used as an indicator of cognitive decline as part of the home interview. Cronbach’s alpha of the MMSE scale was 0.99.

The Center for Epidemiologic Studies Depression Scale (CES-D) [26] was used to assess depressive symptoms during the home interview. The CES-D scale is a widely used self-reported measure in community samples. Its reliability and validity have been demonstrated in heterogeneous samples [27], and in the AGNES sample, Cronbach’s alpha was 0.88. The CES-D assessment includes 20 items in which the respondent rates the frequency of the listed symptoms during the previous week. The validated cut-off score indicating an increased risk for clinically important depressive symptoms in community-living populations is 16 or more of 60 possible points [28].

The Short Physical Performance Battery (SPPB) was used to assess lower extremity performance [29]. Cronbach’s alpha for the SPPB was 0.71. The SPPB includes assessments of standing balance, walking speed over 2.44 m, and timed chair rises (five times). Each task is rated from 0 to 4 points and a total score is calculated (range 0–12) when at least two tests are complete. Higher scores indicate better lower extremity performance.

In the postal questionnaire, the participants were asked whether they took care of a family member who did not manage alone due to illness or disability or needed care for some other reason. If they responded yes, the participants were asked to further define whether they lived with the person they took care of and how frequently they participated in caring for this person, with response options (1) almost around the clock, (2) daily, (3) a few times a week, (4) once a week, (5) two or three times a month, and (6) once a month or less. Those who replied that they took care of their relative at least daily (response options 1 or 2) were categorized as acting as family caregivers.

In the home interview, the participants were asked if they (1) lived alone, (2) with a spouse, (3) with children and/or grandchildren, or (4) with other family members or some others. These were combined into a dichotomous variable living alone (1) vs. living with someone else (2–4). In addition, the participants were asked to report the total number of years of formal education they had attained.

### 2.4. Ethics

This project was approved by the Ethical Committee of the Central Finland Hospital District as a part of the AGNES research project [22]. This study was conducted according to the guidelines for good scientific and clinical practice established by the Declaration of Helsinki. Participants were verbally informed about the research. They also received an information document and provided written informed consent.

### 2.5. Statistical Analyses

Values of descriptive variables with normal distributions were expressed by means and standard deviations (SD). For the descriptive statistics, a statistical comparison between the categories was conducted using analysis of variance (ANOVA). Measures with discrete distributions were expressed as counts with percentages (%) and analyzed via the chi-squared test. The associations between physical activity opportunities and willingness to increase physical activity at different levels of physical activity were analyzed using the multinomial logistic regression model (MLR) and its extension, the three-group multinomial moderation regression model, and we reported the effect size as multinomial odds ratios (mOR). Adjusting variables included missing observations for 3.7% of the subjects and hence the MLR models utilized the maximum likelihood estimator accommodating data based on the missing-at-random (MAR) assumption. The α-level was set at 0.05. Statistical software SPSS for Windows (version 24.0) (IBM Corp., 2016, Armonk, NY, USA) was used for the descriptive analyses [30], and Mplus was used for the multinomial models [31].

## 3. Results

### 3.1. Physical Activity Level

In the total sample (*n* = 962), the proportions of men and women were similar at all physical activity levels. At the low and moderate physical activity levels, 60% were women, and at the high physical activity level, 50% were women (*p* = 0.053). The level of physical activity was different between the age groups (*p* < 0.001). Most often, participants in each age group of 75-, 80-, and 85-year-olds reported their physical activity on the moderate level (75-year-olds 57% vs. 80-year-olds 55% vs. 85-year-olds 48%). Among those with low levels of physical activity, the proportion of 85-year-olds was higher (18% vs. 25% vs. 38%, respectively) while the 75-year-olds were more commonly at the high level of physical activity (26% vs. 20% vs. 14%, respectively).

Those with low physical activity levels were more likely to have decreased cognition (low 13% vs. moderate 7% vs. high 4%, *p* < 0.001) and reported a higher number of depressive symptoms (22% vs. 16% vs. 8%, respectively, *p* < 0.001). Those with low physical activity levels had lowest scores on lower extremity performance (9% vs. 10% vs. 11%, respectively, *p* < 0.001) and also attained the least education (Table 1).

### 3.2. Willingness and Opportunities for Physical Activity

Willingness to increase physical activity differed between physical activity levels (*p* < 0.001). At the low and moderate physical activity levels, two-thirds of the participants wanted to increase their physical activity level. At the high physical activity level, a majority (70%) of the participants did not want to be more active (Table 2). Opportunities for physical activity differed between physical activity levels. Opportunities were most likely to be perceived as poor at the low physical activity level (low 66% vs. moderate 34% vs. high 11%, *p* < 0.001) (Table 2).

### 3.3. Association between Opportunities and Willingness to Increase Physical Activity

Those with poor opportunities for physical activity had approximately 1.8 times higher odds of reporting that they wanted to be a bit more active and approximately 4.9 times higher odds of reporting that they wanted to be a lot more active than of reporting that they did not want to be more active than those with good opportunities (Table 3). The difference between odds ratios was statistically significant (*p* < 0.001).

### 3.4. Association between Opportunities and Level of Physical Activity

Those with poor opportunities for physical activity had approximately 16 times higher odds of being at the low level of physical activity and approximately 4.3 times higher odds of being at the moderate level of physical activity than of being at the high level of physical activity than those with good opportunities. The difference between odds ratios was statistically significant (*p* < 0.001).

### 3.5. Association between Opportunities and Willingness to Increase Physical Activity in Different Physical Activity Levels

Physical activity moderated the association between poor opportunity and willingness to increase physical activity (Table 4). Among those with a low level of physical activity, the association was not statistically significant. At the moderate physical activity level, those who reported having poor opportunities for activity had 1.5 times higher odds (*p* = 0.076) of wanting to be a bit more active and 3.9 times higher odds (*p* < 0.001) of wanting to be a lot more active than not wanting to be more active than those who reported having good opportunities. The difference between odds ratios was statistically significant (*p* < 0.001).

Among the participants who had a high level of physical activity, those with poor opportunities had 3.9 times higher odds of wanting to be a bit more active compared with those with good opportunities and 39 times higher odds of wanting to be a lot more active than not wanting to be more active. The difference between odds ratios was statistically significant (*p* = 0.004). However, only 4.4% of the participants with high levels of physical activity wanted to be a lot more active.

## 4. Discussion

This study showed that those with poorer perceived opportunities for PA were more likely to report willingness to increase physical activity than those reporting good opportunities, while also being more likely to report low physical activity. When analyzed separately for the different physical activity levels, the association between opportunities and willingness manifested among those who had moderate or high physical activity levels. Associations were not found among those with low physical activity. It seems that among older adults who are at least moderately physically active, the wish to increase activity was the most evident when the perceived opportunities for activity were not optimal. Our results indicate that lack of opportunities for physical activity often coincide with willingness to increase physical activity. However, from a health equity perspective, all those who are willing to be physically active should have the opportunity for it regardless of age and functional ability [11].

Many of the participants at the moderate physical activity level perceived that their physical activity level was too low. They reported doing light activities several times per week (for example, home gymnastics, slow walking, or outdoor activities) or activities one or two times per week to the point of some increase in breathing and perspiring (for example, brisk walking, indoor activities, or swimming). We cannot be certain of the specific amount, intensity, and modes of activity. However, presumably not many of them fulfilled the level of physical activity that is recommended as the health-enhancing physical activity level for older adults. It has been reported that only 2.5% of Finnish adults over 75 years meet the recommendation, that is, report meeting the moderate to vigorous intensity physical activity recommendation and at least two times per week of muscle strengthening and/or balance training [14]. It has been reported that when a need is met, it can lead to wellbeing [10]. Although unmet physical activity need can be considered as a felt need [16], it seems that among some older individuals, perceived need of physical activity might reflect not meeting recommended amounts of physical activity sustaining health and functional independence in aging.

Those who were most physically active perceived having good opportunities for physical activity. Yet, one-third of them wanted to be more active. Previously in older adults, better cognition, health, and independence in walking were associated with personal goals related to physical activity [32] and exercise-related goals with higher life-space mobility that is an indication of mobility in daily life [33]. An association between poor opportunities and willingness to increase physical activity was evident among those with high physical activity, showing that unmet physical activity need can be perceived even by those who already have a relatively high activity level. The reasons for perceiving unmet physical activity need among those at moderate and high physical activity levels may be similar. It may be that those who are accustomed to a physically active lifestyle have experienced something that has had a negative effect on their activity engagement and are dissatisfied with the current situation. They may have been used to higher amounts of physical activity or feel that they would like to engage in a specific exercise type that is not available to them at the moment [34]. Reduction in physical activity was previously shown to precede the development of unmet physical activity need over a two-year follow-up [17]. However, with a cross-sectional design, we can only speculate about the conditions leading to the current status.

At the low physical activity level, one-third did not want to be more active. Those at the low level of physical activity more often indicated cognitive decline, had depressive symptoms, and limitations in lower extremity performance. Depressive symptoms have been identified as a barrier to engaging in physical activity among older adults [35] and those living with mild cognitive impairment, early dementia, and more progressed dementia typically report multiple barriers to physical activity [36,37]. Older adults with functional limitations might have settled for their low activity level and may feel that increasing physical activity is impossible for them and thus they do not pursue it. It has been reported that older persons with difficulties in functioning see physical activity as important and value it; however, their preferred modes of activity are walking and taking care of daily domestic chores [38]. On the other hand, some older persons question the usefulness of physical activity in old age and feel that at later stages of life, being sedentary should be allowed [34].

It has been suggested that, for some older persons, the characteristics of physical activity—such as how pleasant or easy they perceive it to be to participate in physical activity—are more important than the health benefits that can be gained through physical activity participation [39]. Moreover, older persons value physical activity that comes from daily activities and may perceive that it is sufficient for them and do not wish to be any more active [34]. Therefore, those with low physical activity levels may be content with their activity levels even though they are aware of other opportunities for physical activity. Moreover, asking only about the willingness to increase the physical activity level may not be adequate for assessing unmet physical activity need. To assess equal opportunities for physical activity, asking about what type of physical activity older persons would like to engage in should be taken into account in future studies.

At the low physical activity level, desire to increase activity levels was observed both among those with good and those with poor opportunities and an association between poor opportunities and willingness to increase activity was not found. The question about opportunities for physical activity was formulated in a way that was intended to produce non-fixed responses, and thus we do not know what the persons considered as opportunities or the lack thereof. For example, we do not know whether they were considering their personal or environmental resources or a combination of these two when assessing their own situation. Nevertheless, the concept of unmet physical activity need was originally developed to describe the discrepancy between perceived need and opportunities to fulfill that need whatever the reasons. Therefore, perception of poor opportunities should be seen as a sign of barriers that stop people from being physically active.

When the need for physical activity is not met, it can decrease an individual’s autonomy and lead to poor health at the individual level, but at the level of the society, it is a sign of inequity. Thus, those who report willingness for physical activity but perceive poor opportunity for it are in need of support. From the ethical standpoint, supporting autonomy is one of the priorities of public health practitioners [40]. It has been suggested that healthy behaviors, such as being more physically active, follows from provision of greater autonomy support and satisfaction of basic needs [15].

From our results, we can conclude that physical activity opportunities are a complex issue that is difficult to capture with a single question. From previous studies that described barriers to physical activity, we know that poor health, fear, negative experiences, an unsuitable environment [41], hills, dangerous cross-roads [42], pain, illness, and poor weather [43] were often reported as barriers to physical activity among older adults and can increase the risk of unmet physical activity need. Environmental features such as having parks, natural areas and good quality walkways nearby home, may provide good opportunities for and facilitate physical activity [44]. In addition, accessibility and affordability of PA can be crucial to some older adults [39], while lack of exercise opportunities specifically targeted to those with dementia is a barrier to their activity [36]. Thus, to understand unmet physical activity need, we need information on the stability of the factors that affect opportunities: is it something that is present all of the time, part of the year, or is it only temporary? It may be that if opportunities for one type of activity are lost, there are still opportunities for something else. The repeatability of our question on opportunities was moderate (Online Resource 1), which may indicate that the question lacks precision or something actually happened that changed their perception on physical activity opportunities over a short period of time.

The strength of this study is that it was based on data collected from a large population-based sample of older adults. A further strength is the questionnaire development that included a pilot study with focus group discussions as well as test–retest reliability analyses of the final questionnaire (Online Resource 1). This study provides new understanding of perceived opportunities and willingness for physical activity among older adults.

Due to the study protocol, older adults with mild or moderate cognitive decline were also included if they were able to participate in the home interview. This could compromise the self-assessment’s reliability. Because the number of participants who perceived their opportunities for physical activity was poor, we had to combine poor with moderate opportunities into one category in the analyses. Therefore, the results cannot be generalized across those with the poorest opportunities for physical activity. It is possible that severe weather conditions, such as extreme heat waves or cold weather, may influence perception of possibilities and on actual level on physical activity, thus influencing unmet need for physical activity. However, in the study area, extreme weather conditions are very rare and during the time of the study, there was normal seasonal variation in weather conditions, and thus this does not provide additional explanation for the results. Unmet physical activity need might manifest differently in other populations with different cultures and appreciations of active aging.

## 5. Conclusions

Older persons who are mostly inactive are likely to report perceiving poor opportunities for physical activity and they express a wish to be more active. However, among them, perceived opportunities are not associated with a perceived need to increase physical activity. Unmet need for physical activity manifests among those who are moderately or even more active. Among them, the wish to increase activity is most evident when the perceived opportunities for activity are not optimal. Considering this new knowledge, increasing equity in physical activity in old age requires provision of support and opportunities at every level of physical activity. For research, we still need to find methods to better assess the individual experience related to willingness to increase, and opportunities for, physical activity, especially among those who are most inactive.

## Figures and Tables

**Table 1:** Descriptive statistics according to physical activity level.

	Low (*n* = 236)	Moderate (*n* = 522)	High (*n* = 204)	*p* Value
Women (%)	59.3	59.5	50.0	0.053 ^2^
Age group (%)				<0.001 ^2^
● 75 (*n* = 439)	18.0	56.5	25.5	
● 80 (*n* = 321)	24.9	55.1	19.9	
● 85 (*n* = 202)	38.1	48.0	13.9	
Family caregiver (%)	12.3	9.4	6.9	0.176 ^2^
Lives alone (%)	47.7	39.0	37.3	0.126 ^2^
Declined cognition (%) (MMSE < 24)	13.4	6.6	4.0	<0.001 ^2^
Depressive symptoms (%) (CES-D > 15)	21.9	15.9	8.0	0.001 ^2^
SPPB mean (SD)	8.5 (2.9)	10.1 (2.1)	11.1 (1.3)	<0.001 ^1^
Length of education (years) mean (SD)	10.8 (3.9)	11.6 (5.0)	12.4 (4.5)	<0.001 ^1^

Note: MMSE = Mini-Mental State Examination, CES–D = The Center for Epidemiologic Studies Depression Scale, SPPB = Short Physical Performance Battery. Tested with ^1^ one–way analysis of variance or ^2^ chi-squared test.

**Table 2:** Willingness to increase and opportunities for physical activity according to physical activity level.

	Low (*n* = 236)	Moderate (*n* = 522)	High (*n* = 204)	*p* Value
	*n* (%)	*n* (%)	*n* (%)	
Willingness to increase PA				<0.001
● A lot more active	48 (20.3)	73 (14.0)	9 (4.4)	
● A bit more active	106 (44.9)	259 (49.6)	53 (26.0)	
● Not more active	82 (34.7)	190 (36.4)	142 (69.6)	
Opportunities for PA				<0.001
● Good	80 (33.9)	344 (65.9)	182 (89.2)	
● Poor	156 (66.1)	178 (34.1)	22 (10.8)	

**Table 3:** Association between opportunities and willingness to increase physical activity (PA) and level of reported physical activity. Results of the joint main effects model for willingness to increase physical activity and level of physical activity predicted by poor vs. good opportunity (*n* = 962).

	mOR (95% CI)	*p* Value (β_1_)	mOR (95% CI)	*p* Value (β_2_)	*p* Value (β_1_–β_2_)
Willingness to increase PA	Willingness to increase PA a bit (vs. not at all)		Willingness to increase PA a lot (vs. not at all)		
● Poor opportunities	1.77 (1.32, 2.37)	<0.001	4.88 (3.21, 7.42)	<0.001	<0.001
● Good opportunities	Ref.		Ref.		
Level of PA	Reporting low PA (vs. high PA)		Reporting moderate PA (vs. high PA)		
● Poor opportunities	16.13 (9.61, 27.08)	<0.001	4.28 (2.65, 6.90)	<0.001	<0.001
● Good opportunities	Ref.		Ref.		

Note: mOR = multinomial odds ratios. Analyzed using the multinomial logistic regression model (MLR) and its extension, the three-group multinomial moderation regression model.

**Table 4:** Association between opportunities and willingness to increase physical activity within physical activity levels. Results of the moderation model with levels of physical activity moderating the effect of poor opportunity (vs. good) on willingness to increase physical activity a bit or a lot (vs. not at all) (*n* = 962).

	Willingness to Increase PA a Bit (vs. Not at All)		Willingness to Increase PA a Lot (vs. Not at All)		
	mOR (95% CI)	*p* Value (β_1_)	mOR (95% CI)	*p* Value (β_2_)	*p* Value (β_1_–β_2_)
Low PA (*n* = 236)					
● Poor opportunities	0.89 (0.94, 1.63)	0.707	1.40 (0.64, 3.06)	0.404	0.240
● Good opportunities	Ref.		Ref.		
Moderate PA (*n* = 522)					
● Poor opportunities	1.46 (0.96, 2.20)	0.076	3.90 (2.21, 6.87)	<0.001	<0.001
● Good opportunities	Ref.		Ref.		
High PA (*n* = 204)					
● Poor opportunities	3.95 (1.39, 11.21)	0.010	38.57 (7.94, 187.36)	<0.001	0.004
● Good opportunities	Ref.		Ref.		

Note: mOR = multinomial odds ratios. Analyzed using the multinomial logistic regression model (MLR) and its extension, the three-group multinomial moderation regression model.

## Data Availability

Due to ethical restrictions, pseudonymized datasets are available only upon request from Professor Taina Rantanen (taina.rantanen@jyu.fi). External collaborators may use data upon agreement on the terms of data use and publication of results.

## Appendix A

### Appendix A.1. Questionnaire Development

The aim is to introduce the questions we used to measure (1) the level of physical activity, (2) willingness for physical activity, and (3) perceived opportunity for physical activity and test their repeatability.

As a starting point, we used questions that were used in a previous research project targeting physical activity in old age [21]. The development of the new questions was started by reviewing existing scales for similar or close topics, keeping in mind the concept of unmet PA need. However, we were not able to identify any questionnaires that would fit our purposes and therefore an expert panel was formed to discuss the topic with the aim of drafting a new questionnaire. An expert panel consisting of members from disciplines of gerontology, psychology, sports education, sport sciences, and physiotherapy met for the first time in September 2016. For a better understanding of the unmet PA need concept, the expert panel produced a pilot questionnaire that was tested in a pilot study in conjunction with the larger AGNES study [22]. A convenience sample for the pilot study was recruited from different contexts (a flu vaccination campaign, a rehabilitation trial, assisted living facilities, the University of the Third Age, and through word of mouth) to guarantee a sufficiently heterogeneous sample. The participants (*n* = 159) responded to a self-administered unmet PA need questionnaire and were asked for written feedback on the questions. In the focus group discussions, participants provided written informed consent prior to the discussion. In the pilot study, the participants consented by returning the questionnaire.

The pilot questionnaire comprised three questions. First, level of physical activity was assessed using a modified version of Grimby’s six-point scale [24,45]. This question was intended to capture all physical activities, from habitual activity to exercise. Willingness to increase physical activity was ascertained using the question “Would you like to increase your level of physical activity?” and opportunity to increase level of physical activity with “How do you perceive your opportunities for physical activity?”

The respondents’ feedback showed that specific examples of physical activity were needed for questions related to willingness and opportunities for physical activity. The participants were unsure whether the questions prompted exercise or habitual activity. Thus, examples of physical activities that described both habitual and exercise activity (for example, activities during daily errands, walking to a store, cycling, physical exercises, etc.) were added. The questions about willingness for physical activity and opportunities for physical activity were easily understood.

After the pilot study, two focus group sessions were conducted to discuss the respondents’ understanding of the concept of physical activity and ascertain how they perceived the pilot questionnaire’s questions. Focus group members were recruited from the University of the Third Age and through word of mouth. Both focus group sessions had eight participants.

The focus group discussions were led by an expert panel member while another researcher took notes on the discussion. First, the focus group members were introduced to the concept of unmet PA need, and asked to give their opinion about the three questions’ relevance. Then all of the focus group participants completed the questionnaire, and its contents were discussed. The participants provided additional examples of physical activity to add to the questionnaire that were added as in the final version.

At the end, the focus group participants decided unanimously that the pilot questionnaire drafted by the researchers was easy to understand and respond to.

The questionnaire was in Finnish. The pilot study, focus group discussion, and data collection were conducted in Finnish among native Finnish speakers.

### Appendix A.2. Questionnaire

Level of physical activity was assessed with the question “Which of the following options best describes your current physical activity? Include exercise and habitual physical activity, such as walking to a store and cycling. Choose one option.”

(1) I do not do more activities than is necessary to perform daily chores.

(2) I do light activities 1–2 times per week (for example, home gymnastics, slow walking, or outdoor activities).

(3) I do light activities several times per week (for example, home gymnastics, slow walking, or outdoor activities).

(4) I do activities 1–2 times per week to the point of some increase in breathing and perspiring (for example, brisk walking, indoor activities, or swimming).

(5) I do activities several times per week to the point of some increase in breathing and perspiring (for example, brisk walking, indoor activities, or swimming).

(6) I do activities that lead to quite a large increase in breathing and perspiring or participate in competitive sports.

In the analyses, alternatives 1 and 2 (low), 3 and 4 (moderate), and 5 and 6 (high) were combined due to low response frequencies in the most extreme categories.

Willingness to increase physical activity was assessed with the question “Would you like to increase your level of physical activity?” with response options:

(1) Yes, I would like to be very much more active than I currently am.

(2) Yes, I would like to be much more active than I currently am.

(3) Yes, I would like to be a bit more active than I currently am.

(4) No, I would not like to be more active than I currently am.

In the analyses, alternatives 1 and 2 (a lot more active) were combined due to low response frequencies in the most extreme categories. Alternatives 3 (a bit more active) and 4 (not more active) were used as independent categories.

Opportunity for physical activity was assessed with the question “How do you perceive your opportunities for physical activity? Include exercise and habitual physical activity, such as walking to a store or cycling.” Response options were: (1) very good, (2) good, (3) moderate, (4) poor, and (5) very poor/not possible. In the analyses, alternatives 1 and 2 (good) as well as 3, 4, and 5 (poor) were combined due to low response frequencies.

### Appendix A.3. Reliability

#### Appendix A.3.1. Test–Retest Procedure

Test–retest analysis was used to determine the reliability of the final questions over time. The replicability sample (*n* = 134) comprised the participants who had responded to the AGNES postal questionnaire and participated in the laboratory assessments at the research center between October to December 2017. All of the participants who took part in the test–retest study were 75 years of age.

The participants answered the questions on a sheet of paper, first as part of the AGNES postal questionnaire, and then during their visit at the research center as a separate questionnaire. The median time between the first and second rounds was 12 (interquartile range 9–15, range 2–46) days.

#### Appendix A.3.2. Statistical Analyses

The test–retest repeatability for the individual items was evaluated by calculating the weighted kappa, an extension of the generalized concordance correlation coefficient for categorical data [46].

#### Appendix A.3.3. Test–Retest Results

The participants in the replicability sample, that is, those who completed both questionnaires in test–retest phase (*n* = 134) were all 75 years old and 57% (*n* = 77) were women. The weighted kappa of the individual questions between the test and retest was 0.68 (*p* < 0.001) for the PA level, 0.60 (*p* < 0.001) for willingness to increase PA, and 0.45 (*p* < 0.001) for opportunities for PA, indicating moderate similarity in the participants’ responses to all three questions on both occasions. The study sample in the test–retest reliability analyses consisted only of 75-year-old participants whereby the validity of the questions used in other age groups remains unclear.
